# A Belladonna Case—Why Not Mercurius?

**Published:** 1893-03

**Authors:** W. E. Ledyard

**Affiliations:** San Francisco, Cal.


### A BELLADONNA CASE—WHY NOT MERCURIUS?

W. E. LEDYARD, M. D., SAN FRANCISCO, CAL.

May 6th, 1892.—A youth, aged sixteen, presented himself for treatment with the following symptoms:

*Extensive swelling of* the *gum* of the lower jaw, on the left side, adjacent to a decayed tooth, the swollen gum *extending over* the latter. First noticed the swelling six days previously. He *applied Chloroform* and the swelling subsided, but three days subsequently, the gum again began to swell.

Here it appears to me we see the vital force acting more powerfully than the *suppressing Chloroform,* and thus overcoming the suppression.

The patient expressed the pain as "*jumping,*" *a severe pain that comes and goes;* the affected part was *extremely painful to the touch; relieved by warmth; aggravated by cold air;* saliva keeps flowing from the mouth; can only partially open the mouth; tongue coated yellow on the dorsum; *sour* taste in the mouth; sleepless, on account of the pain.

The above looks very much like a *Mercurius* case; however, *Bell.* was given in the 200th potency, a dose, dry, with instructions to repeat the same, *in solution,* hourly, for five doses, in twenty-four hours, if the improvement had then ceased.

In forty-eight hours the patient reported that the relief was speedy and decided, and that the swelling had almost disappeared.

Let us differentiate between Mercurius and Belladonna.

Merc. and Bell. both have:

1. Painful swelling of the gum,
2. Salivation, and
3. Bad taste in the mouth.

On examining the swollen gum, we notice that *the swelling extends over the tooth,* almost covering the latter, while under Mercurius we read: Gums *painful to touch,* swollen, *receding from the teeth;* whitish edges, *bleeding,* with a fetid odor from the mouth.

In the above case, the *sensitiveness to touch* was extreme. This *extreme* sensitiveness we find under *Bell.* Aggravation from touch, while present under Merc., is not nearly so marked.

The *Bell.* pains "*come and go*" suddenly, and the taste is sour, as with the patient.

*Merc.* has a sweetish, slimy, salty, etc., taste. It also has aggravation from warmth of bed, and from cold or warm things.

In our case we have amelioration *from warmth*, and aggravation from cold air.

The choice of the remedy was decided in favor of *Bell.*, on account of

1. The great aggravation from touch.
2. The amelioration from warmth.
3. The swelling of the gum *covering* the teeth, and not receeding from them, as in Merc., etc.
4. The sour taste, and especially
5. The suddenness with which the pains came and went.

### Case II.—Arsenicum in Ovaritis.

June 26th, 1890.—Married woman, aged forty-three, light complexion, the mother of seven children, had a miscarriage two weeks previous to above date. This was preceded by profuse hemorrhage, which commenced about *midnight.*

She was attended by a professed homœopath, but not a Hahnemannian.

Complained of *burning* in the right ovary; much *worse at night*, with *burning* in right thigh; a terrible *weakness* from the waist down, *on attempting to walk;* has to lie down most of the time. The pain is *excited* or *aggravated* by mental *anxiety.* Ars.$^{500}$, dose, at once, dry. Repeat in the morning, after a bad night. Sent *three* powders.

July 4th.—Received a letter, saying: "The medicine (Ars.$^{500}$) seemed to do a great deal of good, *at first, removing the sore feeling from the waist down*, without affecting the ovary trouble.

"There is *restlessness before midnight;* hard to get to sleep; when I do so, I am all right until morning.

"I am seldom bright and well in the morning. Intense *burning in right ovary*, which extends down the *inner side of the right thigh*, nearly as far as the knee; very *weak* and nervous, and good for nothing. Very constipated."

July 5th.—Ars.$^{500}$, in solution, every two hours, for twenty-four hours, or until relieved.

July 28th.—The Ars. ameliorated very decidedly until a few days ago. Ars.$^{40m}$, in solution, one hour, for three doses.

Some time after, the patient informed me that the above symptoms were completely relieved.

In the pathogenesis of this wonderful antipsoric we find as characteristics:

1. Anxiety.
2. Restlessness.
3. Burning, especially in the abdomen, and very frequently referred to the *right side* of the abdomen.
4. The *weakness*, which is extreme. The patient doesn't realize her weakness *until she begins to move.* In the above case this was expressed by the words "on attempting to walk."
5. Aggravation at night.

### Case III.—Lycopodium in Diphtheria.

April 9th.—A woman, married, but not living with her husband; aged twenty-five, light complexion, blue eyes, circular, scaly eruption on face, probably syphilitic.

Had used a *gargle*, and had also *painted the throat*, before we were called in.

The following symptoms were elicited:

"Bad cold" two months; throat very sore, worse at night; worse on *right* side; relieved by lying on the back; profuse salivation; large yellow exudation on right tonsil; tonsils swollen, worse on the right; tonsils and fauces purplish; frequent swallowing; tongue thick; dark-yellow coating on dorsum; pain in throat from talking; pain in throat, better *by pressure of the tongue against the teeth;* darting pains in right throat when swallowing; *burning in throat*, aggravated *by cold and* ameliorated *by hot drinks;* very "nervous"; no thirst; fetor of breath; hoarseness for two months; warm sweat after flushing; menses four days overdue; constipation; sedentary habits.

Probably I should have waited until the alteration in the throat-symptoms produced by the gargle and painting had disappeared. However, I did nothing of the kind, but on account of the *burning in throat* aggravated *by cold water*, I gave Canth.$^{200}$, at first, a dose, dry.

Four hours later, there being no improvement, I administered the same in solution, every half-hour, giving six doses.

After the first dose, *the burning was slightly relieved*, but the throat was still as sore.

Here, I confess to a great mistake in repeating.

The *relief from warm drinks* is mostly *after* swallowing.

Flush and sweat simultaneously.

Sweats especially from feet up to waist.

Smarting in throat aggravated by drinking cold water.

Urine dark-red and thick.

Has had the *womb painted with Iodine*, for "inflammation." *Since then, leucorrhœa.*

Now, cotton-like clots come away while urinating.

10th.—Burning and smarting in throat much less; salivation has decreased; not so much pain in throat when talking; slept better; exudation on tonsil disappearing; awoke with general sour-smelling sweat; pulse, 84.

Sensation in right tonsil as though an abscess were about to burst.

April 11th.—Last night restless; dreams of money, drinking, and smoking (she does drink, and smokes cigarettes, which I, of course, prohibited).

During the night, sweating profusely.

Throat still very sore, with slight burning and smarting, *now slightly on left side.*

Saliva runs from the mouth when she lies down, and, during sleep, is offensive (musty), staining yellow.

Throat seemed to be filling up during the night.

Talking causes a tired aching at the root of the tongue.

Can't lie on left side on account of fullness at præcordium; formerly, *the same symptom* prevented lying on the right side.

Exudation, now thin and white, with angry, fiery-red border on right arch of palate; yellowish-white coating on dorsum of tongue.

1. Throat troubles passing from right to left: Lyc.
2. Amelioration from warm drinks: Lyc.
3. Membrane white; Lyc. and many others.
4. Fetor—Lyc. and many others.

April 11th.—At 3.45 P. M.; Lyc.$^{200}$, dose, dry.

April 12th.—Very much better.

April 13th.—Still improving; appetite "splendid."

April 14th.—Slight relapse from going out yesterday, contrary to orders.

Lyc.$^{200}$, half-hour, in solution, for six doses—completed the cure.

I couldn't find the symptom: "Pain in throat better by pressing the tongue against the teeth." What remedy has this symptom?

In this case, did the *slight relief to the burnings* that followed the first dose of the solution of Canth. show that that was the remedy? Although the burning and smarting were less and the exudation was disappearing, the improvement was so much more rapid and the general condition of the patient so much better, after the first dose of Lyc. that it seems right to conclude that Lyc. was *the* remedy from the first.

### Case IV.—Whooping-Cough and Pneumonia, Cured by Cuprum Metallicum.

November 22d, 1891.—Girl, aged five and one-half years; thin, delicate, aggravation from two to three hours after going to bed until four A.M. Cough with whoop for one week; coughs in sleep sometimes; cough sometimes compels her to sit up; cough followed by crying; cough excites pain in throat; cough with vomiting of mucus; cough excited by crying; does not care to play; sits or lies about all day; cough with lachrymation; cough wakes her; cough excited by moving; holds the breath a long time during the paroxysm of coughing. Râles at base of left lung posteriorly; cough with rattling in chest.

Gave several remedies without relief until the 27th, when I elicited the symptom,

*Cough relieved by drinking cold* water.

This, with the rattling in the chest decided me to give *Cupr-met.*$^{200}$ in solution, a dose after every *severe* paroxysm.

November 29th.—Very much better. Repeat as before. She received no more medicine, the paroxysms gradually becoming less frequent and less severe until they disappeared.

It is always best to stop the remedy *when the paroxysms of coughing become less severe*, even if the cough should continue in a mild form for weeks. The reason for pursuing this course is the fact that the cough is the *result* and not the cause of the disease. The same thing holds good with regard to any cough and many other morbid conditions. Continuing the remedy in such cases will almost invariably cause a return of the severe symptoms.

### Case V.—Arnica in Whooping-Cough.

December 1st, 1891.—An older sister of Case IV.

Paroxysms of whooping-cough very severe:

1. With anxiety.
2. With vomiting of mucus.
3. — ecchymosis of conjunctiva of left eye.
4. — fear and loss of breath.

No relief until the 7th, when the symptoms were:

1. Crying before paroxysm: Arn., Bell.
2. — after —: Arn., Bell., Caps., Cina.
3. Pain in chest during —: Arn., Bell.

These, with the ecchymosis decided in favor of Arnica, which was given in the 200th potency, in solution, after a severe paroxysm, with decided relief.

### Case VI.—Arsenicum in Ovaritis.

Woman, aged forty-one; light complexion; married; seven children; the youngest being five years old.

*Burning*, intense and constant, *in right ovarian region* aggravated by walking, ameliorated partially by lying down.

The burning sensation extends to the knee; is much *worse at night*, and is accompanied by a dull aching with heaviness. *Weakness* great from the waist down; *compels her to lie down* the greater part of the time.

*Anxiety* (concerning a member of her family) or any *exertion of the body* will cause a return of the burning and other symptoms. Dreadfully nervous and irritable; very weak and good for nothing; utterly discouraged and miserable, with a frequent feeling that *this trouble is my death-warrant.*

Sensation of something *heavy* and *egg-shaped* in right ovarian region, with occasional *drawing* pains; *cramps* and strange sensation in the right thigh.

*Restless before midnight*, lying awake a long time.

In the *morning* seldom bright and well. "Obstinately constipated."

From the 6th of April, 1889, to the 28th of July, 1890—*i. e.*, a period of over fifteen months—the above symptoms were relieved by *Arsenicum*, ranging in potencies from the 200th to the 40m, as follows:

April 6th.—*Ars.*$^{200}$, in solution, every hour, for *three* doses. This relieved until

April 26th.—*Ars.*$^{200}$, in solution. Sent a few powders with directions to take one when the burning is very severe. Was relieved again until

May 2d.—*Ars.*$^{1000}$, one dose, dry.

June 26th, 1890.—A period of *thirteen months* had elapsed since the last prescription.

Two weeks before the above date had a *miscarriage*, which was preceded by a profuse hemorrhage, commencing about *midnight.* Was the retnrn of the ovary trouble caused by the extra pressure on the system during the pregnant state?

*Arsenicum*$^{500}$, dose, dry.

July 5th—*Arsenicum*$^{500}$, in solution, every *two* hours for twenty-four hours, or until relieved.

July 28th.—*Arsenicum*$^{40m}$, a dose, in solution, *night and morning*, until *three* doses are taken.

July 21st, 1892.—To my knowledge, there has been no return of the above trouble worth mentioning, although the patient is now far advanced in pregnancy with the eighth child.

*Comments.*—Among the *remedies especially affecting the right ovary,* we find Æsculus-hippocastanum, Apis, Arsenicum, Argentum-nitricum, Belladonna, Bryonia, Ferrum, Glonoine, Graphites, Hamamelis, Iodium, Lachesis, Lac-caninum, Lycopodium, Natrum-muriaticum, Palladium, Rhus-toxicodendron, Saccharum-lactis, etc.

Of these there are only two that produce a sensation of *burning* in the right ovary, viz., *Arsenicum* and *Belladonna.*

As *Belladonna does not have* the *mental symptoms* which are present in the above case, *it* cannot possibly be the remedy ; and just as surely as *Arsenicum has those symptoms,* just so surely will it and no other be the indicated remedy.

In prescribing, we usually begin with the 200th, giving one dose, dry.

If this fail to relieve within a few hours (or a few days, in chronic, comparatively painless cases), we then give *the same potency in solution,* usually every half-hour or every hour, according to the urgency of the case, until about five doses have been taken, or until relieved.

If this be followed by relief, we *do not repeat as long as the amelioration continues.* When it ceases, we administer *a higher potency,* in the same manner as before—*i. e.,* first dry, and afterward, if necessary, in solution.

It may be profitable to compare the above symptoms with the pathogenesis of *Arsenicum.*

*Arsenicum* has, as *mental characteristics:* Anxiety, restlessness, exhaustion, and fear of death—all of which we find in our case.

It also has, "Thoughts of death and the incurability of her complaint."

The *Arsenicum* patient is "Irritable, discouraged, restless, and vexed about trifles."

The patient does not know how *weak* she is until she attempts to move, or to exert herself in any way. In the example before us, *any effort was followed by great weakness, compelling her to lie down.*

The inclination to lie down is very marked under *Arsenicum.*

*Burning* is exceedingly characteristic of this remedy, especially burning in the abdomen.

*Burning in* region of *right ovary,* and circumscribed swelling, we also find, as well as "Intense burning or tensive pain in the ovary, with great restlessness." Among its sensations are, as in the case before us, crampy pains, drawing and heaviness. *Aggravation at night* is likewise common to the remedy and the disease under discussion.

"Like cures like," and consequently *Arsenicum* being the simillimum, removed the symptoms and cured the patient.

